# Current Progress in Evolutionary Comparative Genomics of Great Apes

**DOI:** 10.3389/fgene.2021.657468

**Published:** 2021-08-11

**Authors:** Aisha Yousaf, Junfeng Liu, Sicheng Ye, Hua Chen

**Affiliations:** ^1^CAS Key Laboratory of Genomic and Precision Medicine, Beijing Institute of Genomics, Chinese Academy of Sciences, Beijing, China; ^2^China National Center for Bioinformation, Beijing, China; ^3^University of Chinese Academy of Sciences, Beijing, China; ^4^CAS Center for Excellence in Animal Evolution and Genetics, Chinese Academy of Sciences, Kunming, China

**Keywords:** evolutionary comparative genomics, natural selection, structural variations, new genes, great apes

## Abstract

The availability of high-quality genome sequences of great ape species provides unprecedented opportunities for genomic analyses. Herein, we reviewed the recent progress in evolutionary comparative genomic studies of the existing great ape species, including human, chimpanzee, bonobo, gorilla, and orangutan. We elaborate discovery on evolutionary history, natural selection, structural variations, and new genes of these species, which is informative for understanding the origin of human-specific phenotypes.

## Introduction

**FIGURE 1:**
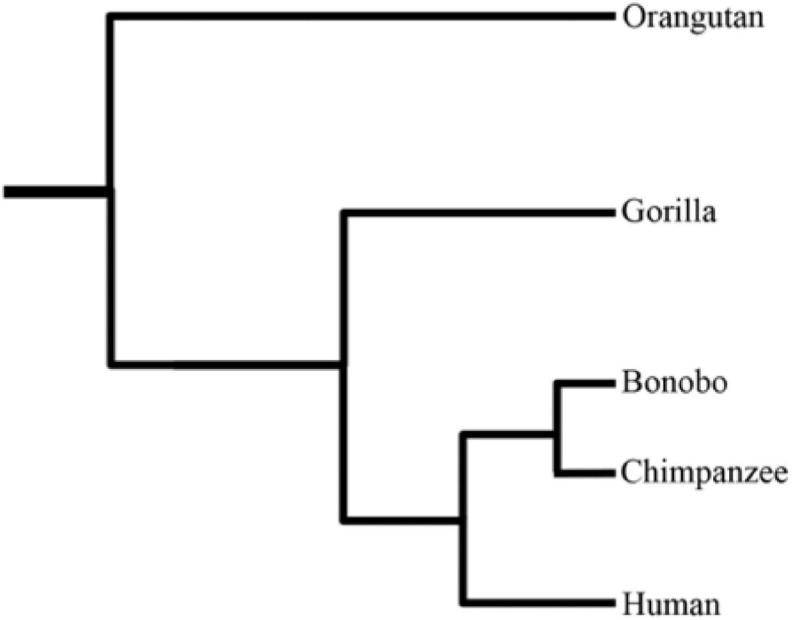
The phylogenetic tree of great ape species (Prado-Martinez et al., 2013).

The pronounced upsurge in modern sequencing technologies during the recent past has led to immense genomic data across a wide range of species. This spearheaded efforts to better understand the divergent species’ genome architecture, and unfold the potential adaptation mechanisms. Recently, interest has grown to provide enhanced insights into the genetic diversity of primates using comparative and population genomic data. Being *Homo sapiens*’ closely related cousins, non-human primates (NHPs) provide a stepping stone for better understanding the evolutionary origins of human-specific traits. Moreover, they can also serve as models for studying the genetic basis of human disease phenotypes. Humans markedly differ from the closely related NHPs on various grounds, including, but not limited to, brain size, cognitive capacities, social behavior, language, craniofacial features, bipedalism, hairless skin, and advanced tool usage (Gagneux and Varki, 2001; Carroll, 2003). Natural selection is deemed responsible for species’ adaptations to changing environments. During recent years, a plethora of studies focused on pinpointing the natural selection signatures in humans and their closely related cousins to understand the genetic basis of modern humans’ adaptation and their evolutionary uniqueness. Herein, we briefly review the recently conducted evolutionary comparative genomic studies of great apes regarding their evolutionary history, natural selection, new genes (originated in humans), and structural variation landscape. Figure 1 illustrates the phylogenetic relationship among great ape species.

## Genome Assemblies

The first genome sequence of chimpanzee was assembled in 2005, which was compared with the human genome for generating the catalog of genetic differences found among both species (The Chimpanzee Sequencing and Analysis Consortium, 2005). The Sumatran orangutan draft genome has been assembled in 2011 and compared to the other primates (Locke et al., 2011). Prüfer et al. (2012) sequenced and assembled the bonobo genome in 2012 for studying the evolutionary relationship with chimpanzee and human genomes. Although the short-read sequencing has been widely used for whole-genome assembly due to the decreasing cost and increasing throughput, the repetitive DNA sequences may make the assembly incomplete (Gordon et al., 2016). Recently developed long-read sequencing technologies, single-molecule real-time (SMRT) sequencing by PacBio sciences, and Oxford Nanopore Technologies’ (ONT) nanopore sequencing have proved promising in generating the highly contiguous genomes. Using SMRT sequencing, Gordon et al. (2016) presented the long-read sequence assembly of gorilla genome with a novel assembly algorithm, which can use long (>10 Kbp) sequence reads. Kronenberg et al. (2018) generated the genome assemblies of human, chimpanzee, and orangutan using SMRT long-read sequencing with over 65-fold coverage. Mao et al. (2021) reported a new high-quality bonobo genome assembly using the long-read PacBio platform with 74-fold sequence coverage. The details of recently assembled genomes of great apes can be found in Table 1. The availability of high-quality whole-genome sequences of extant great ape species provides an avenue to conduct highly refined comparative genomics research in great ape lineages.

**TABLE 1:** A summary of whole-genome sequencing in great apes.

**Species**	**Platform**	**Genome coverage**	**Assembly size (Gbp)**	**Number of contigs**	**Contig N50 (Mbp)**	**Scaffold N50 (Mbp)**	**References**
Chimpanzee	PacBio RSII	>65.0×	2.99	5,037	12.42	53.10	Kronenberg et al., 2018
Bonobo	PacBio RSII	74.0×	3.02	4,975	16,580	68,247	Mao et al., 2021
Gorilla	PacBio RSII	74.8×	3.08	16,073	9.56	23.14	Gordon et al., 2016
Orangutan	PacBio RSII	>65.0×	3.04	5,813	11.07	98.47	Kronenberg et al., 2018

## Evolutionary History

### Genomic Introgression

Previous studies based on molecular data report that humans and chimpanzees diverged ∼5–7 million years ago (Mya) (Chen and Li, 2001; Brunet et al., 2002). However, this estimate is incompatible with the divergence estimates drawn based on fossil data, which is ∼6–7 Mya. The study led by Langergraber indicates that human–chimpanzee split likely occurred at least 7–8 Mya (Langergraber et al., 2012). Recently, Besenbacher et al. (2019) yielded divergence estimates by extrapolating the non-human mutation rates over the great apes’ phylogenetic tree and dated humans and chimpanzees split to 10.6 Mya. The difference between the two genomes is ∼1%. By analyzing the divergence among hundreds of DNA sequences between the two genomes, Osada and Wu (2005) inferred that the speciation history between human and chimpanzee could not be the same for coding and intergenic regions. The results suggest that the speciation of human and chimpanzee may not be allopatric speciation. The divergence time between sequences, which belong to two closely related species, shows variation throughout the genome because of the polymorphism and the stochastic fluctuation. The exponential distribution can be used to describe the variation. However, gene flow occurring between the species elicits increased disparity in the divergence time between sequences. By comparing neutral autosomal loci between human and chimpanzee, the data unveiled gene flow in ancestral lineages leading to humans and chimpanzees millions of years ago (Yang, 2010; Liu et al., 2015).

Genetic admixture events among chimpanzee subspecies and bonobos were also investigated based on the genome-wide statistics and site frequency spectrum (SFS)-based modeling (de Manuel et al., 2016). A demographic model based on SFS supports multiple events of genetic admixture between chimpanzees and bonobos. According to this model, gene flow from bonobos into the central and eastern chimpanzees occurred approximately 200,000 and 550,000 years ago. This subsequently spread into Nigeria–Cameroon as a result of admixture between Nigeria–Cameroon and central and eastern chimpanzees. A more recent gene flow event occurred between bonobos and central chimpanzees approximately >200,000 years ago. Furthermore, Kuhlwilm et al. (2019) also detected the signatures of archaic gene flow between bonobos and extinct great ape populations and found the evidence for archaic admixture between bonobos and divergent great ape lineage. They compared the landscapes of introgression in humans and bonobos and showed that *SERPINA11* and *SERPINA9*, which are related to adaptive immunity, are in the longest introgression regions.

### Demographic History

Chimpanzees (*Pan troglodytes*) and bonobos (*Pan paniscus*), which together form the *Pan* genus, are the closest extant relatives of the humans, which experienced a bottleneck out of Africa (Li and Durbin, 2011). They have an estimated divergence time of 1–2 Mya (Prüfer et al., 2012). Despite the very short split time, both species markedly differ in terms of their demographic histories and also exhibit lineage-specific differences for several behavioral and phenotypic traits, which make them an interesting subject for comparative genomic studies. Chimpanzees are distributed across Africa ranging from central Africa to eastern and western regions of Africa. Radiation among chimpanzee subspecies began approximately 600,000 years ago (Hoelzel, 2016). Although substantial differences exist in the effective population sizes (*Ne*) of chimpanzee subspecies, all chimpanzee subspecies have greater *Ne* except western chimpanzees and harbor higher genetic diversity than their sister species bonobo (de Manuel et al., 2016). Bonobos are found in the Democratic Republic of the Congo (central Africa); central chimpanzees (*Pan troglodytes troglodytes*) bound their distribution to the west, and the eastern chimpanzee (*Pan troglodytes schweinfurthii*) to the north and south. Bonobos have experienced population bottlenecks and have a smaller long-term *Ne* (11,900–23,800) (Prado-Martinez et al., 2013; de Manuel et al., 2016). The rapid decline in the bonobo population during the recent past has rendered them among the endangered species.

In gorillas, there are four sub-species: eastern gorillas, western gorillas, cross-river gorillas, and mountain gorillas. The four gorilla species have different demographic histories over the past 200,000 years (Prado-Martinez et al., 2013). The western gorillas have the largest historical *Ne* while the historical *Ne* of the eastern gorillas is the smallest. Additionally, the mountain gorillas experienced severe population bottleneck and are a critically endangered subspecies in central Africa (Xue et al., 2015). Orangutans are the most distant relatives of humans among the extant great apes and have two sub-species: Sumatran orangutans and Bornean orangutans, which were designated as distinct species in 2001 (Xu and Arnason, 1996; Groves, 2001). They have an estimated divergence time of 1 Mya and also experienced severe population bottleneck, which makes it urgent to protect these endangered species (Prado-Martinez et al., 2013). However, Nater et al. (2017) show a new orangutan sub-species, which is an isolated population from Batang Toru and is distinct from Sumatran and Bornean populations. Their analyses revealed that the divergence time between Batang Toru populations and the ancestral populations of Sumatran orangutans and Bornean orangutans is ∼3.38 Mya.

## Natural Selection

Many tests designed to detect the natural selection signals identify the genomic loci that show departure from a standard neutral model as probable targets of natural selection. Usually, dN/dS (the non-synonymous/synonymous rate) ratios provide a simplistic measure of selective pressures experienced by a genomic loci, with dN/dS >1 as an indicator of positive selection at the protein level (McDonald and Kreitman, 1991; Yang and Nielsen, 2002, 2008). In comparison, it is difficult to detect the adaptive evolution of non-coding DNA because of the lack of natural benchmark. Siepel et al. introduced methods to test natural selection on non-coding DNA (Gronau et al., 2013; Huang et al., 2017). The method analyzes polymorphism of a single population with sequences of one or several outgroup species, and can distinguish the effects of strong positive, strong negative, and weak negative selection on the basis of their influence on polymorphism and divergence patterns. The aforementioned methods provide an opportunity to detect the natural selection signal across the whole genome.

In earlier studies of *Pan* genus, the effectiveness of purifying selection has been analyzed and the results showed the correlation between past *Ne* and the efficacy of natural selection (Bataillon et al., 2015; Cagan et al., 2016). Cagan et al. (2016) presented the first global map of natural selection in great apes based on genome-wide information by combining several neutrality tests. They found that most signatures of positive selection are species-specific, such as the signature driven by the gene *AMY2B* related to diet. Daub et al. (2017) focused on four branches of the primate tree and identified the biological pathway signals of adaptation in the primate phylogeny. They found that the selection signals in the candidate pathways are elicited by different genes in the different branches during the course of primate evolution. Bertranpetit et al. tested signatures of adaptive introgression in chimpanzees based on SFS (Nye et al., 2018). They found the evidence of subspecies-specific adaptations in introgressed regions, which are involved in processes such as the male reproduction in central chimpanzees, the immune system in eastern chimpanzees, and the nervous system in Nigeria–Cameroon chimpanzees. Han et al. (2019) analyzed the largest available dataset of *Pan* populations and reported the demographic history and purifying selection as the underlying factors for existing genetic variation in *Pan* species. They also found that the small past *Ne* correlates with a larger number of deleterious alleles and the genes enriched with bonobo-specific non-synonymous changes are related to age at menarche in humans. Based on a new bonobo genome assembly, Mao et al. (2021) identified some novel genes and found that most of the novel genes showed selective sweeps in bonobo, such as *DIRC1*, *GULP1*, and *ERC2*.

Recently, Zhao et al. (2019) developed a method, HDMKPRF (aka high-dimensional MKPRF), which is built in the Poisson random field framework developed by Sawyer and Hartl (1992), and is an extension of their MKPRF method from two species to multiple species (Bustamante et al., 2001). They constructed a spatial–temporal landscape of natural selection signatures that occurred across the species’ evolutionary history (Zhao et al., 2019). The method pools information over multiple gene loci and gains power over the traditional single-gene-based MK test to detect positive selection signals (Zhao et al., 2019). Given the high efficiency of the method, genome-wide selection scans across great apes except of bonobos using the HDMKPRF method, which jointly analyzes the within-species polymorphism and cross-species divergence data, provide comprehensive insights into the lineage-specific selection across multiple species. The authors found that the positively selected genes identified in the human lineage are enriched in gene expression regulation pathways, immune system, and metabolic pathways.

## Structural Variations

Structural variants (SVs) are an important class of variants that are at least 50 bp in size. SVs have increased capacity to rearrange genomic content than small-scale insertions/deletions (indels; <50 bp) or single-nucleotide variants (SNVs). Owing to their size and abundance, SVs potentially have increased capacity to affect gene expression, shape up genome evolution, and impact phenotypes (Perry et al., 2008; Weischenfeldt et al., 2013). A large number of studies aimed at detecting structural variations in humans and NHPs have contributed a great deal to our understanding of SV abundance and their functional impact. These studies used a wide array of technologies to discover SVs (Gokcumen et al., 2013; Kuderna et al., 2017; Catacchio et al., 2018; Kronenberg et al., 2018).

Catacchio et al. (2018) performed genome-wide comparisons between human, great ape, and macaque genomes and detected 156 putative inversions belonging to 136 human gene loci. These inversions showed considerable variation in their size ranging from 103 Kbp to 91 Mbp. They also found 67 inversions in either one or multiple primates, with 36 inversion breakpoints overlapping with 81 human genes. In addition, they also unveiled the evolutionary history of these genomic inversions to investigate functional differences among primate genomes. The genes impacted by these inversions showed enrichment in several functional categories, including transport proteins, DNA-binding proteins, receptors (G protein-coupled receptors and olfactory receptors), and drug metabolism (cytochrome P450). However, the importance of these genes in bringing about phenotypic differences among humans and other primates remains unknown. Kronenberg et al. (2018) employed long-read sequencing technology to generate high-quality assemblies of human, chimpanzee, and orangutan genomes and identified all SVs >50 bp in size within great ape genomes. They identified a total of 17,789 SVs specifically fixed in human lineage (fhSVs) that impact protein-coding regions as well as regulatory regions with some of these deletions related to human-specific phenotypes. For instance, a large human-specific deletion SV (65 Kbp) was detected in two genes, *FADS1* and *FASD2*, which are also positive selection targets in modern humans and are implicated in fatty-acid biosynthesis (Ameur et al., 2012). This fhSV may have functionally contributed to differential dietary habits of great apes (from herbivores to omnivores) during the course of their evolution (Ye et al., 2017). Notably, two fhSVs were also found in *WEE1* and *CDC25C* genes, which are cell cycle regulators and act as ultrasensitive antagonists. These genes are expressed in radial glia and, as a result of increased cell division therein, are speculated to underlie neocortical expansions in human lineage.

In a recent study, Soto et al. (2020) performed long-range and -read sequencing (using optical nanopore sequencing) of two chimpanzee individuals (belonging to the *Pan. troglodytes verus* subspecies) in order to characterize their SV landscape. A total of 124 novel SVs with size ≥10 kb (including 88 deletions and 36 inversions) were identified in chimpanzees. The study highlighted that 56 genes impacted by putative chimpanzee-specific SVs could lead to chimpanzee-specific phenotypic traits. Deletion SVs showed overrepresentation in “sensory perception of smell” and “G-protein coupled receptor signaling pathway.” Furthermore, some of the genes impacted by SVs appeared likely targets of natural selection, which is suggestive of the significance of SVs in impacting the chimpanzee adaptation during the course of evolution. Comparing the bonobo genome to the other great apes’ genomes, Mao et al. (2021) identified 22,868 bonobo-specific SVs with size >50 bp (including 7,082 deletions and 15,786 insertions), among which there are 1,965 fixed deletions and 3,604 fixed insertions.

## New Genes

New genes are often implicated in the emergence of lineage-specific traits and serve as an important resource for evolutionary innovation. For decades, the birth of new genes was attributed to the modification of preexisting genes (Ohno, 1970). In the recent past, studies also reported the birth of new genes from scratch, by DNA- or RNA-mediated mechanisms (Kaessmann, 2010; Betrán, 2015). These mechanisms include gene fusion, horizontal gene transfer, virus domestication, retroduplication, and *de novo* gene origination. *De novo* genes also known as “motherless” or “orphan” genes may constitute a significant proportion (up to 10%) of all new genes (Zhang et al., 2010). Interestingly, *de novo* protein-coding genes have also been found in humans similar to other species (Wu et al., 2011). Wu et al. (2011) identified 60 protein-coding genes of *de novo* origin in human lineage implicating a higher-than-expected rate of *de novo* gene birth. Some of these genes were highly expressed in the cerebral cortex pertaining to their involvement in cognitive capacities.

Cai and Petrov (2010) classified human and chimpanzee orthologous protein-coding genes into distinct age classes (i.e., young and old genes) based on their distribution breadth across the phylogenetic tree. Old genes are broadly distributed compared with young genes, which exhibit restricted phylogenetic distribution and appear in closely related species. Notably, the study unveiled frequent non-synonymous polymorphism experienced by young genes contrary to old genes. These findings suggested that stronger purifying selection acts upon old genes while young genes experience weaker purifying selection and hence evolve faster (Cai and Petrov, 2010). Furthermore, the study also highlighted disparities in the deleterious mutation burden of two gene classes with older genes being abundant in slightly deleterious mutations than younger genes.

Xie et al. (2012) conducted genome-wide identification of *de novo* protein-coding genes that were specific to hominoids and unveiled the precise origin timing of these genes in vertebrate phylogeny. Furthermore, comparative transcriptomic profiling was also performed in human, chimpanzee, and rhesus macaque to address how these *de novo* protein-coding genes came into being. Notably, most of these protein-coding genes were found to be originated from non-coding RNAs (Xie et al., 2012). The study presented a “semi-product” model of *de novo* gene birth and evolution.

Focusing on new genes, Shao et al. (2019) created an integrated online database, named GenTree. GenTree gleans age estimations from multiple gene-dating methods and incorporates the functional genomic data, gathered from Human Protein Atlas, for new genes. By performing genome-wide comparison of the existing age estimation methods, the study unveiled synteny-based pipeline (SBP) as the most suitable method for dating recently duplicated genes. However, for dating ancient genes, protein-family-based methods proved promising. Shao et al. (2019) also curated a list of 254 SBP-dated primate-specific protein-coding genes (PSGs) with different levels of protein evidence. Classifying these PSGs into co-expressing modules spotlighted the functional bias of these PSGs. Notably, PSGs were predominantly involved in male reproduction, defense response, mother–fetus interaction, and brain development. These findings highlighted that PSGs are recruited to processes under strong selection pressure or show biased recruitment in organs with rapidly evolving pathways, for instance, an expanded brain and placenta. Taken together, PSGs were contemplated as a group of genes potentially contributing to primate-specific phenotypic evolution.

## Conclusion

Over the past years, the successful completion of the human genome project and the release of whole-genome sequences of NHPs led to a plethora of studies highlighting the most conspicuous differences between humans and their closely related phylogenetic cousins. Natural selection is deemed responsible for conferring unique characteristics to a species or within species trait divergence that includes behavioral, cognitive, dietary, and phenotypic differences, among others. Furthermore, the natural selection signatures identified in humans and their closely related cousins help reveal the mechanism of human evolution. In addition, the systematic discovery of SVs by comparing improved great ape genome assemblies provides enhanced insights into the evolution of structural variations and their contribution to lineage-specific phenotypes during the course of great apes’ evolution.

Although a large number of fossils of humans have been found, they are mainly used to calibrate the divergence time in the studies of evolutionary comparative genomics of great apes. However, ancient DNA that can be extracted from the fossil has great potential advantages in the evolutionary comparative genomics research (Pickrell and Reich, 2014; Hofreiter et al., 2015). Firstly, ancient DNA can be used to track migration and natural selection directly. Secondly, ancient DNA makes it possible to observe genetic variation patterns of extinct species directly. Finally, ancient DNA allows us to study history interactions between extinct species and modern species. Therefore, ancient DNA from archaic hominins, such as Neanderthals and Denisovans, can serve as more closely related outgroups and provide novel insights into the evolution of humans (Slatkin and Racimo, 2016; Yang and Fu, 2018; Zhang and Fu, 2020). Furthermore, how to take advantage of ancient DNA in the studies of evolutionary comparative genomics of great apes will be a promising direction for future research.

## Author Contributions

HC designed the manuscript. AY, JL, SY, and HC wrote the manuscript. All authors contributed to the article and approved the submitted version.

## Conflict of Interest

The authors declare that the research was conducted in the absence of any commercial or financial relationships that could be construed as a potential conflict of interest. The reviewer DG declared a past co-authorship with one of the authors SY to the handling editor.

## Publisher’s Note

All claims expressed in this article are solely those of the authors and do not necessarily represent those of their affiliated organizations, or those of the publisher, the editors and the reviewers. Any product that may be evaluated in this article, or claim that may be made by its manufacturer, is not guaranteed or endorsed by the publisher.
